# Inhibition of P-Selectin and PSGL-1 Using Humanized Monoclonal Antibodies Increases the Sensitivity of Multiple Myeloma Cells to Bortezomib

**DOI:** 10.1155/2015/417586

**Published:** 2015-10-11

**Authors:** Barbara Muz, Feda Azab, Pilar de la Puente, Scott Rollins, Richard Alvarez, Ziad Kawar, Abdel Kareem Azab

**Affiliations:** ^1^Cancer Biology Division, Department of Radiation Oncology, Washington University in Saint Louis School of Medicine, Saint Louis, MO 63108, USA; ^2^Selexys Pharmaceuticals, 840 Research Parkway, Suite 510, Oklahoma City, OK 73104, USA

## Abstract

Multiple myeloma (MM) is a plasma cell malignancy localized in the bone marrow. Despite the introduction of novel therapies majority of MM patients relapse. We have previously shown that inhibition of P-selectin and P-selectin glycoprotein ligand-1 (PSGL-1) play a key role in proliferation of MM and using small-molecule inhibitors of P-selectin/PSGL-1 sensitized MM cells to therapy. However, these small-molecule inhibitors had low specificity to P-selectin and showed poor pharmacokinetics. Therefore, we tested blocking of P-selectin and PSGL-1 using functional monoclonal antibodies in order to sensitize MM cells to therapy. We have demonstrated that inhibiting the interaction between MM cells and endothelial and stromal cells decreased proliferation in MM cells and in parallel induced loose-adhesion to the primary tumor site to facilitate egress. At the same time, blocking this interaction *in vivo* led to MM cells retention in the circulation and delayed homing to the bone marrow, thus exposing MM cells to bortezomib which contributed to reduced tumor growth and better mice survival. This study provides a better understanding of the biology of P-selectin and PSGL-1 and their roles in dissemination and resensitization of MM to treatment.

## 1. Introduction

Multiple myeloma (MM) is a plasma cell malignancy located mainly in the bone marrow (BM), characterized by continuous dissemination of cancer cells [1, 2]. Accumulating evidence indicates that egress of MM cells from one site of the BM to a new site is a complex process that involves cellular and acellular interactions with endothelial cells, stromal cells, soluble growth factors, and extracellular matrix. Molecular mechanisms of cell adhesion and cell trafficking and thus metastasis in MM have been intensively investigated [3, 4]. The interactions of MM cells with the BM microenvironment play a crucial role in cell survival, cell trafficking, and drug resistance in MM; and interrupting these interactions enhances MM cells sensitivity to chemotherapy [3–7].

Selectins (CD62) are cell surface lectin-like adhesion molecules which bind sugar polymers and are involved in lymphocyte extravasation, especially during inflammation and cancer metastasis [8]. Selectin family consists of E-selectin, L-selectin, and P-selectin, expressed on endothelium, leukocytes, and platelets, respectively [8]. When endothelium is activated, P-selectin travels to the cell surface and can bind to ligands expressed on both leukocytes and cancer cells. The selectins and ligands interact rapidly in order to facilitate tethering, followed by rapid dissociation to enable rolling on the endothelium and ultimately cell extravasation [9]. P-selectin glycoprotein ligand-1 (PSGL-1, CD162) is the best characterized ligand for all three types of selectins and is expressed on myeloid, lymphoid, and dendritic cells [10]. PSGL-1 undergoes posttranslational modifications which are required to bind selectins and are similar for binding P-selectin and L-selectin [11]. PSGL-1 has especially high affinity for P-selectin on intact leukocytes compared to other ligands and is essential for adhesion to P-selectin [12, 13].

During cancer metastasis, cell adhesion and cell migration are frequently malfunctioning. Since cancer cells mimic leukocytes exploiting selectin-dependent mechanisms to extravasate, there is a growing interest in blocking selectins and their ligands in inflammation, tumor progression, and metastasis [14–16]. In solid tumors, it was demonstrated that absence or blocking of P-selectin with antibody decreased tumor cell adhesion and metastasis in rat lungs [17], gastric cancer in mice [18], and colorectal cancer [19].

Both P-selectin and PSGL-1 were also suggested as new targets in MM [6, 20, 21]. Expression of PSGL-1 was reported in normal plasma cells, with higher levels of PSGL-1 indicating plasma cell differentiation [6, 22]. PSGL-1 was shown to be highly expressed in MM biopsies and MM cell lines [5, 6, 23], and PSGL-1 gene expression increased in the course of MM progression [6]. Another study performed on MM biopsies demonstrated a significant correlation between the degree of PSGL-1 expression and the Durie-Salmon stage; thus PSGL-1 could be used as a diagnostic marker in MM [21]. It was previously demonstrated that knocking down PSGL-1 with siRNA in MM cells delayed tumor initiation* in vivo* [6]. Moreover, blocking selectins with pan-inhibitor GMI-1070 in MM mouse model in combination with bortezomib inhibited tumor growth during treatment and delayed tumor progression after halting the therapy significantly improving mice survival [6]. However, this inhibitor was previously shown to be a potent inhibitor of E-selectin and a nonpotent inhibitor of P-selectin, with high concentrations needed to inhibit P-selectin [24]. The necessity of using very high concentrations of GMI-1070 to achieve inhibition of P-selectin-mediated interactions of MM cells with the BM microenvironment limits the possibility to translate it into clinical settings. Thus, there is an urgent need to use novel, specific, and potent P-selectin/PSGL-1 interaction inhibitors.

In this study, we focused on the role of blocking P-selectin and PSGL-1 to inhibit MM progression and dissemination using specific humanized blocking antibodies for P-selectin and PSGL-1. We tested MM cell adhesion and proliferation* in vitro*, as well as MM cells extravasation, homing, tumor growth, and mice survival* in vivo*. These studies emphasize the importance of targeting P-selectin and PSGL-1, in combination with bortezomib, in the context of BM microenvironment, as a promising therapy for MM patients.

## 2. Materials and Methods

### 2.1. Cell Culture

The MM cell lines (MM1.s and H929, mycoplasma-negative) were obtained from American Type Culture Collection (ATCC, Rockville, MD). MM cell lines were cultured in RPMI-1640 media (Corning CellGro, Mediatech, Manassas, VA) supplemented with 10% fetal bovine serum (FBS, Gibco, Life Technologies, Grand Island, NY), 2 mmol/L of L-glutamine, 100 U/mL Penicillin, and 100 *μ*g/mL Streptomycin (CellGro, Mediatech, Manassas, VA). Human umbilical vein endothelial cells were purchased from Lonza, Allendale, NJ. Human stromal cells were obtained from MM patients' BM biopsies depleted of CD138-positive myeloma plasma cells. Endothelial cells were cultured in endothelial cell growth media (EGM-2, Lonza) and stromal cells were cultured in 20% FBS Dulbecco's Modified Eagle's Medium (Corning CellGro, Mediatech, Manassas, VA) containing L-glutamine, Penicillin/Streptomycin. Cells were cultured at 37°C (5% CO_2_) in the NuAire water jacket incubator (Plymouth, MN).

### 2.2. Animals

SCID-beige mice (females, 8-week old) and Balb/C mice (females, 9-week old) were obtained from Charles Rivers Laboratories (Wilmington, MD). Approval for these studies was obtained from the Ethical Committee for Animal Experiments at Washington University in St. Louis Medical School.

### 2.3. Effect of SelG1 and SelK2 on MM Cell Adhesion and Proliferation

The humanized monoclonal antibodies anti-P-selectin (SelG1) and anti-PSGL-1 (SelK2) were obtained from Selexys Pharmaceuticals (Oklahoma City, OK). For adhesion assay, endothelial and stromal cells (3 × 10^3^ cells per well in 96-well plate) were incubated with SelG1 (10 *μ*g/mL) and MM cells prelabeled with calcein-AM of final concentration 1 *μ*g/mL (Invitrogen, Life Technologies, Grand Island, NY) were incubated with SelK2 (10 *μ*g/mL). MM cells were applied to unlabeled endothelial cells or stromal cells for 1 hr, nonadherent cells were aspirated, and adherent cells were measured by detecting the fluorescent intensity signal using fluorescent reader (excitation/emission = 485/520 nm). For proliferation assay, H929 prelabeled with DiD (Invitrogen) were cultured alone, with endothelial or stromal cells, and were treated with or without bortezomib (Selleck Chem, Houston, Texas), in presence or absence of SelG1 and SelK2 antibodies, and cell proliferation was determined by flow cytometry.

### 2.4. Effect of SelG1 and SelK2 on MM Cell Extravasation and Homing

MM1.s labeled with calcein-AM were injected intravenously (IV) into Balb/C mice (3 × 10^6^ cells/mouse) creating 3 groups: (1) mice treated with anti-mouse P-selectin antibody injected intraperitoneally (IP) the day before (*n* = 3); (2) mice treated with anti-mouse PSGL-1 antibody (rat anti-mouse CD162 antibody, catalog number 557787, BD Pharmingen, San Jose, CA) injected IP the day before and MM1.s treated with SelK2 antibody (anti-human PSGL-1) for 1 hr prior to the injection (*n* = 3); or (3) untreated MM1.s (*n* = 3). 50 *μ*L of blood was collected from the portal vein at 50 min after injection, red blood cells were lysed with a buffer (BioLegend, San Diego, CA), and the circulating calcein-AM-positive MM cells were counted by flow cytometry. The presence of MM cells in the circulation signified retention, or in other words the absence of these cells in the circulation signified extravasation of the MM cells. After the last blood aspiration, mice were sacrificed; mononuclear cells were isolated from femurs, washed, and analyzed by the flow cytometry. The number of calcein-AM-positive cells was analyzed in each mouse and reflected the number of MM cells which homed to the BM.

### 2.5. Tumor Progression and Survival Study

MM1.s cells were genetically engineered to express green fluorescent protein (GFP) and luciferase (Luc), as described previously [25]. In the first experiment, human MM1.s-GFP-Luc cells were injected into 24 SCID mice IV at a concentration of 2 × 10^6^ cells per mouse and allowed to grow for 3 weeks. The mice were then divided randomly into 3 groups (8 mice/group) and treated as follows: (1) vehicle control which received PBS as vehicle; (2) anti-mouse P-selectin antibody (5 mg/kg) (rat anti-mouse CD62P antibody, catalog number 553741, BD Pharmingen, San Jose, CA) to inhibit P-selectin in the mouse stroma and endothelium; and (3) SelK2 and anti-mouse PSGL-1 (5 mg/kg) (catalog number 557787, BD Pharmingen) to inhibit PSGL-1 on human MM cells and in the mouse microenvironment, respectively. Tumor progression was monitored by bioluminescence imaging (BLI) once a week for 4 weeks (week 3 = time 0). In the second experiment, human MM1.s-GFP-Luc cells were injected into 32 SCID mice IV at a concentration of 2 × 10^6^ cells per mouse and allowed to grow for 2 weeks. The mice were then divided randomly into 4 groups (8 mice/group) and treated as follows: (1) vehicle control; (2) bortezomib alone (1 mg/kg); (3) bortezomib (1 mg/kg) + anti-mouse P-selectin antibody (5 mg/kg); and (4) bortezomib (1 mg/kg) + SelK2 and anti-mouse PSGL-1 (5 mg/kg). Tumor progression was monitored by BLI twice a week for 4 weeks (week 2 = time 0). In both experiments, the vehicle, bortezomib, and antibodies were injected IP twice a week. Bortezomib and antibodies were administered IP sequentially twice a week. Tumor progression was followed twice a week using bioluminescence imaging. Survival of mice was followed every day by investigator, with no blinding.

### 2.6. Statistical Analysis

**Figure 1:**
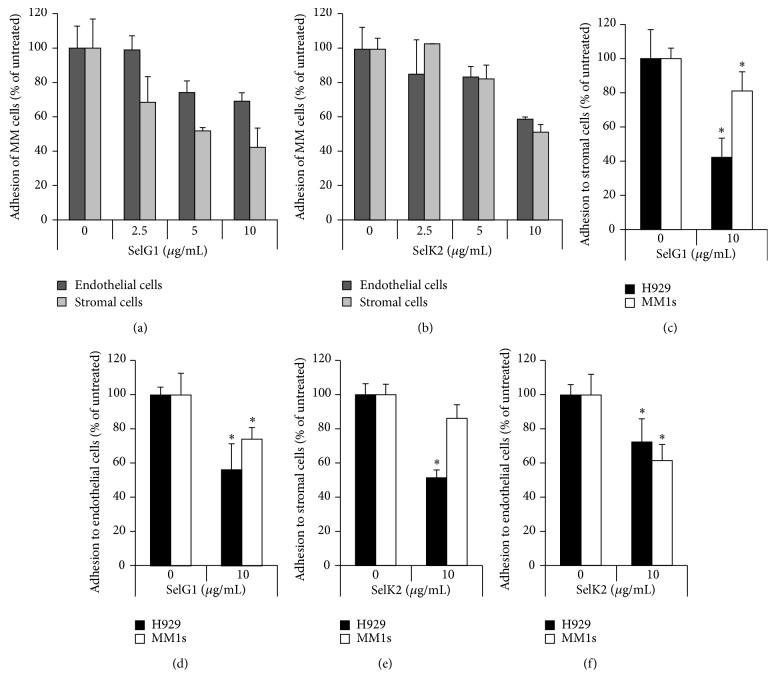
P-selectin and PSGL-1 regulate adhesion of MM cells to endothelial and stromal cells* in vitro*. Endothelial and stromal cells were treated with increasing concentrations of SelG1 antibody (2.5, 5, and 10 *μ*g/mL) followed by adhesion of H929 or MM1.s cells labeled with calcein-AM. MM cell adhesion was assessed as a signal of adherent calcein-AM-positive MM cells measured by fluorescent reader and normalized to untreated cells (a). Likewise, H929 or MM1.s cells labeled with calcein-AM were incubated with increasing concentrations of SelK2 antibody (2.5, 5, and 10 *μ*g/mL) and plated on untreated endothelial and stromal cells, and the cell adhesion was measured as above (b). Stromal (c) and endothelial cells (d) were treated with SelG1 (10 *μ*g/mL) for 1 hr followed by plating MM1.s and H929 labeled with calcein-AM, and the adhesion was assessed as a signal of adherent calcein-AM-positive MM cells measured by fluorescent reader and normalized to untreated cells. Likewise, MM1.s and H929 were treated with SelK2 (10 *μ*g/mL) for 1 hr and plated on stromal (e) or endothelial cells (f), and the cell adhesion was measured as above. Values were considered significant for ^*∗*^ *p* < 0.05.

**Figure 2:**
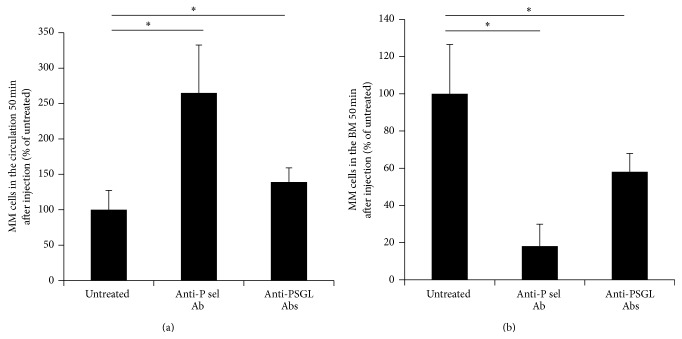
Blocking P-selectin and PSGL-1 decrease extravasation and decrease homing of MM cells to the BM* in vivo*. The effect of P-selectin- and PSGL-1-blocking antibodies on the number of circulating MM cells detected as calcein-AM-positive MM cells detected by flow cytometry at 50 minutes of blood aspiration after injection (a). The effect of blocking P-selectin and PSGL-1 on MM cell homing to the BM shown as the number of calcein-AM-positive cells detected in the BM, analyzed by flow cytometry, and normalized to untreated cells (b). Results are depicted as mean ± s.d. and statistical significance was analyzed by student *t*-test (*n* = 5 mice per group). Values were considered significant for ^*∗*^ *p* < 0.05.

**Figure 3:**
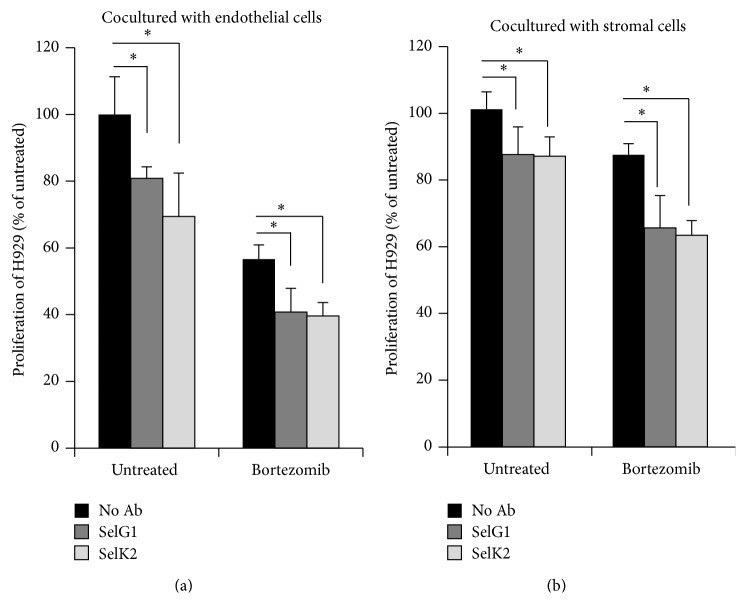
P-selectin and PSGL-1 affect proliferation of MM cells cocultured with endothelial and stromal cells* in vitro*. The effect of SelG1 antibody (10 *μ*g/mL) used on endothelial and stromal cells and SelK2 (10 *μ*g/mL) used on H929 cells on MM cell proliferation, with or without bortezomib (5 nM) treatment, analyzed by flow cytometry and normalized to untreated cells. Values were considered significant for ^*∗*^ *p* < 0.05.

**Figure 4:**
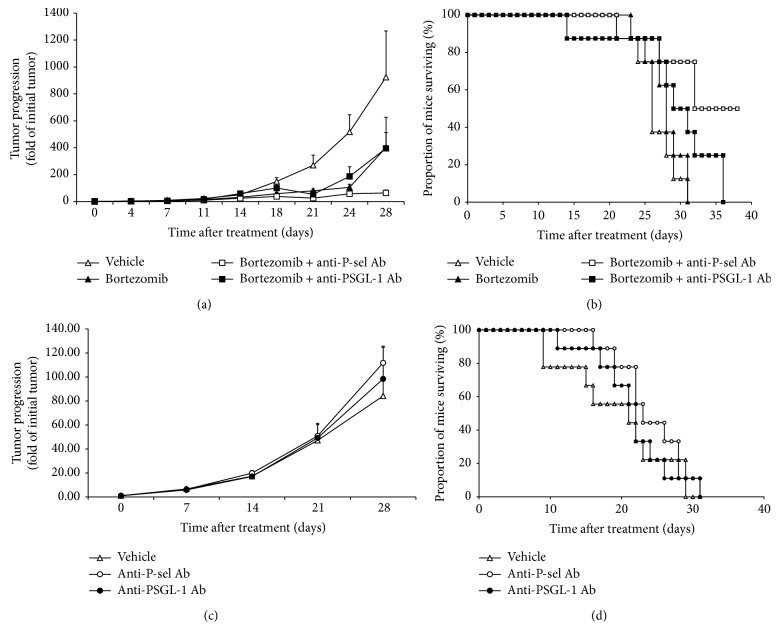
Inhibition of P-selectin in combination with bortezomib decreases tumor size and improves survival in MM mouse model* in vivo*. The effect of P-selectin and PSGL-1 inhibition on sensitivity to bortezomib of the MM-bearing mice. SCID mice (*n* = 8 per group) were injected with MM1.s-GFP-Luc and tumor growth was determined by bioluminescence imaging (BLI). In the first experiment (c and d), tumor was allowed to grow for 3 weeks. The mice were then divided into 3 groups: (1) vehicle control; (2) anti-mouse P-selectin antibody (5 mg/kg); and (3) SelK2 and anti-mouse PSGL-1 (5 mg/kg). Tumor progression was monitored by BLI once a week for 4 weeks (week 3 = time 0). In the second experiment (a and b), tumor was allowed to grow for 2 weeks and then the mice were divided randomly into 4 groups: (1) vehicle control; (2) bortezomib alone (1 mg/kg); (3) bortezomib (1 mg/kg) + anti-mouse P-selectin antibody (5 mg/kg); and (4) bortezomib (1 mg/kg) + SelK2 and anti-mouse PSGL-1 (5 mg/kg). Tumor progression was monitored by BLI twice a week for 4 weeks (week 2 = time 0). Tumor progression was detected shown as region of interest (ROI) normalized to initial tumor size in each group; the statistical significance was assessed by student *t*-test. The statistical significance was present between groups: vehicle versus bortezomib (*p* = 0.0025), vehicle versus P-sel + bortezomib (*p* = 0.0023), vehicle versus PSGL-1 + bortezomib (*p* = 0.01), bortezomib versus P-sel + bortezomib (*p* = 0.0018), and P-sel + bortezomib versus PSGL-1 + bortezomib (*p* = 0.0172) (a). Survival of mice was followed 40 days after starting the treatment and depicted as Kaplan-Meier curve. The *p* values were calculated from the chi-squared test for independence. The statistical significance was present between groups: vehicle versus P-sel + bortezomib (*p* = 0.005) and bortezomib versus P-sel + bortezomib (*p* = 0.01) (b).

The* in vitro* experiments shown on Figures 1 and 3 were performed in quadruples and replicated independently two more times. Results are shown as mean ± s.d. The* in vivo* experiments, also depicted as mean ± s.d., were analyzed using student *t*-test (Figure 2) or chi-squared test (Figure 4) for independence for statistical significance, with the data meeting the assumption of the tests such as normal distribution. Variation within each group was equally variant and similar between the groups that were statistically compared. Values were considered significantly different for *p* value less than 0.05.

## 3. Results

### 3.1. P-Selectin and PSGL-1 Regulate Adhesion of MM Cells to Endothelial and Stromal Cells

First, we tested different concentrations (2.5, 5.0, and 10 *μ*g/mL) of SelG1 (Figure 1(a)) or SelK2 (Figure 1(b)) on MM cell adhesion to endothelial and stromal cells and we found a dose-dependent effect of these monoclonal antibodies. Next, we examined the adhesion of MM cells (H929 and MM1.s) to stromal and endothelial cells. We found that after blocking P-selectin using a single concentration of SelG1 (10 *μ*g/mL) on stromal cells H929 adhesion was decreased by 60% and MM1.s by 20% (Figure 1(c)); or on endothelial cells H929 adhesion was decreased by 43% and MM1.s by 23% (Figure 1(d)). Likewise, after blocking PSGL-1 on MM cells using a single concentration of SelK2 (10 *μ*g/mL), H929 cell adhesion was decreased by 50% and MM1.s by 12% in coculture with stromal cells (Figure 1(e)), or H929 cell adhesion was decreased by 28% and MM1.s by 40% in coculture with endothelial cells (Figure 1(f)).

### 3.2. Blocking P-Selectin and PSGL-1 Decrease Extravasation and Decrease Homing of MM Cells to the BM* In Vivo*

To examine the role of P-selectin and PSGL-1 interaction on extravasation and homing of MM cancer cells to the BM* in vivo*, we injected MM cells labeled with calcein-AM and detected the number of calcein-AM-positive cells both in the blood and the BM samples 50 minutes after injection, indicating extravasation and homing, respectively. In mice pretreated with anti-mouse P-selectin antibody, MM cells displayed delayed extravasation with approximately 2.7-fold more MM cells still present in the circulation, whereas, in mice pretreated with anti-mouse PSGL-1 antibody and anti-human SelK2, there were 1.4-fold more MM cells in the circulation compared to untreated mice at time 50 minutes (Figure 2(a)). In case of homing, pretreatment with anti-mouse P-selectin antibody decreased the number of MM cells that had homed to the BM by 82%, whereas in mice pretreated with anti-mouse PSGL-1 antibody and SelK2 homing was decreased by 42%, compared to untreated mice (Figure 2(b)).

### 3.3. P-Selectin and PSGL-1 Affect Proliferation of MM Cells Cocultured with Endothelial and Stromal Cells

It was demonstrated before that the interaction between BM microenvironment and MM cells contributes to drug resistance [4]. Here we investigated the effect of inhibiting the interaction between P-selectin and PSGL-1 on MM cell proliferation detected by flow cytometry. The stroma and endothelial cell-induced proliferation of H929 was decreased using SelG1 or SelK2. Similar effects were observed when combining the antibodies with bortezomib, in which SelG1 or SelK2 enhanced the effect of bortezomib on H929 proliferation when cocultured with endothelial cells (Figure 3(a)) and stromal cells (Figure 3(b)).

### 3.4. Inhibition of P-Selectin in Combination with Bortezomib Decreases Tumor Size and Improves Survival in MM Mouse Model* In Vivo*

Finally, we studied the effect of blocking P-selectin and PSGL-1 on MM tumor progression in MM xenograft mouse model in combination with bortezomib. MM tumors were established in SCID mice and one week before bortezomib initiation, two groups of mice were pretreated twice a week with P-selectin and PSGL-1 antibodies. The combination of P-selectin antibody with bortezomib inhibited tumor growth significantly, compared to vehicle control and bortezomib alone treated mice. On the other hand, combination of PSGL-1 antibody with bortezomib delayed tumor growth to similar extent as bortezomib alone (Figure 4(a)). Survival study revealed that mice treated with P-selectin antibody (but not PSGL-1 antibody) in combination with bortezomib had significantly prolonged survival compared to other groups (Figure 4(b)). Moreover, we found that inhibiting P-selectin or PSGL-1 alone does not influence tumor progression (Figure 4(c)) and mice survival (Figure 4(d)), compared to untreated mice.

## 4. Discussion

MM is characterized by continuous dissemination of cancer cells throughout the BM [1, 2]. During metastasis in MM, adhesion of cancer cells to vascular wall requires the presence of P-selectin on the endothelium and stroma and PSGL-1 on cancer cells [6]. It was demonstrated that PSGL-1 is highly expressed on MM cells and regulates adhesion and cell trafficking in MM; these interactions involve both endothelial and BM stroma cells which express high levels of P-selectin [6]. PSGL-1 was previously suggested as a novel target for immunotherapy in MM using monoclonal antibody, where anti-PSGL-1 antibody increased cell death of MM cells in a time- and dose-dependent manner [26]. Loss-of-function study and a small molecule pan-selectin inhibitor GMI-1070 demonstrated that PSGL-1 and P-selectin regulate the activation of integrins, adhesion, and proliferation, as well as downstream signaling. The anti-pan-selectin treatment using GMI-1070 sensitized MM cells to bortezomib* in vitro* and* in vivo*, controlling growth, dissemination, and drug resistance of MM in the context of the BM microenvironment. However, low specificity of GMI-1070 to P-selectin and its pharmacokinetic profile with a very short half-life may hold up further usage of this drug [6].

In the present study, we examined the effect of potent humanized monoclonal antibodies targeting P-selectin and PSGL-1, and we confirmed that both antibodies decreased MM cell adhesion to endothelial and stromal cells. Since the MM cells in the presence of SelG1 and SelK2 were less adhesive* in vitro*, we tested the blocking effect of these antibodies on cell extravasation* in vivo* and we found that inhibiting the interaction of P-selectin and PSGL-1 prevented MM cell extravasation and increased their circulation time* in vivo*. At the same time, blocking P-selectin and PSGL-1 significantly decreased the capability of cancer cells to home to the BM* in vivo*. These results are in agreement with our previous studies showing that inhibition of the interaction using siRNA downregulation of the genes or the small molecule inhibitor reduced the adhesion of MM cells to endothelial and stromal cells* in vitro* and prevented extravasation and homing to the BM* in vivo* [6].

Next, we studied the effect of P-selectin and PSGL-1 antibodies on proliferation of MM cells when cocultured with endothelial or stromal cells. Results showed that inhibition of the interaction of MM cells with stromal and endothelial cells using the anti-P-selectin (SelG1) and the anti-PSGL-1 (SelK2) antibodies reversed the stromal and endothelial cell-induced proliferation of MM cells. Similarly, the antibodies increased the sensitivity of MM cells to bortezomib when these were cocultured with stromal and endothelial cells. These results confirm that blocking the P-selectin/PSGL-1 sensitizes MM cells to chemotherapy.

We then tested the effect of blocking P-selectin on the BM microenvironment and blocking PSGL-1 on MM cells on proliferation and drug resistance of MM cells* in vivo*. The antibodies alone did affect neither tumor growth nor mice survival; this is in agreement with our previous findings that GMI-1070 alone did not induce any delay in tumor progression* in vivo*. However, we found that inhibition of the interaction between PSGL-1 and P-selectin using the humanized antibodies, SelG1 and SelK2, induced sensitization of MM cells to bortezomib, increased the survival of animals with MM, and delayed tumor progression. The combination of bortezomib with the anti-P-selectin antibody SelG1 was more effective than the combination of bortezomib with the anti-PSGL-1 antibody SelK2. The possible mechanism for delayed tumor growth could be prolonged circulation and exposure of cancer cells in the peripheral blood to bortezomib due to increased cell retention and reduced cell homing. In addition, it was demonstrated previously that PSGL-1 and selectins are involved in macrophage-mediated drug resistance in MM; by blocking PSGL-1 in MM cells with antibody or by silencing PSGL-1 with shRNA using lentiviral vector, MM cells were resensitized to melphalan when cocultured with macrophages, which was driven by Erk1/2 pathway activation and c-myc upregulation [23].

In conclusion, our results demonstrate that inhibition of P-selectin/PSGL-1 axis using humanized monoclonal antibodies, SelG1 and SelK2, is promising as a treatment for MM and that these antibodies were potent (only 5 ug/mL was needed) with a very good pharmacokinetics (antibodies were injected twice a week only). The use of the antibodies disrupted the interaction between MM cells and BM microenvironment, decreased proliferation and adhesion of MM cells* in vitro*, and delayed tumor growth and extended survival in MM xenograft mouse model. This data provides a basis for future clinical trials for sensitization of refractory MM patients to therapy by blocking the P-selectin/PSGL-1 axis using the humanized monoclonal antibodies SelG1 and SelK2.
